# Diagnosis of sinusoidal obstruction syndrome: can biopsy be the key?

**DOI:** 10.1007/s00277-023-05445-6

**Published:** 2023-09-23

**Authors:** Oliver Kriege, Andreas Kreft, Beate Hauptrock, Pascal Wölfinger, Matthias Theobald, Eva-Maria Wagner-Drouet

**Affiliations:** 1 Center for Cellular Immunotherapy and Stem Cell Transplantation, Third Medical Department, Hematology and Oncology, University Cancer Center Mainz (UCT), Langenbeckstr 1, 55131 Mainz, Germany; 2 Department of Pathology, University Medical Center Mainz, Langenbeckstr 1, 55131 Mainz, Germany

Dear Editor,

Veno-occlusive disease/sinusoidal obstruction syndrome (VOD/SOS) is a rare, often devastating systemic endothelial disease typically developing within days/weeks after hematopoietic cell transplantation (HCT) or other endothelial damage (e.g., non-HCT chemotherapy, liver transplantation). Characterized by hyperbilirubinemia, thrombocytopenia, painful hepatomegaly, ascites, ≥ 5% weight gain, and right upper quadrant pain, VOD/SOS can rapidly progress to multiorgan failure (MOF) and death if left untreated [1]. Early, precise diagnosis and treatment are mandatory but often complicated and delayed by lack of clear diagnostic criteria, with symptoms overlapping graft-versus-host disease (GvHD), sepsis, and other causes of MOF. EBMT diagnostic criteria [2] may overcome these challenges only partly.

By presenting a severe and fatal case of late-onset VOD/SOS, we highlight the difficulties to find the correct diagnosis and show a possible solution. A 31-year-old female patient underwent HCT in our institution for an acute myeloid leukemia (FLT3-ITD+, t[8;21] translocation) after 7+3 induction and cytarabine-based consolidation therapy combined with midostaurin. After 1 year in complete remission, rising RUNX1-RUNX1T1 levels led to preemptive HCT with a 10/10 HLA-matched unrelated donor. The HCT comorbidity index score was 1 (hepatic, formerly hepatitis b); major VOD/SOS risk factors were present such as donor type, myeloablative conditioning, and iron overload (ferritin 8270 ng/ml pre-HCT).

The conditioning regimen comprised busulfan/cyclophosphamide and ATG plus GvHD prophylaxis with methotrexate/cyclosporine; VOD/SOS prophylaxis was made with ursodeoxycholic acid, no low-dose heparin was used. During inpatient treatment, capillary leak occurred under ATG and afterwards, bilirubin peaked at day 13 (8.3 mg/dl) after with no further VOD/SOS features and improved spontaneously. Granulocyte recovery occurred on day 16, with patient discharge on day 24 without further complication.

At the first outpatient visit (day 29), the patient showed platelet recovery and fever. She was hospitalized and treated with piperacillin/tazobactam. On day 33, a sudden rise in creatinine (2.3 mg/dl) and bilirubin (11.2 mg/dl, doubling in 48 h) occurred. Transaminases increased to 6 times upper limit of normal, with positive procalcitonine, fever, and abnormal coagulation plus a weight increase of 5–10%. Liver ultrasound showed acute cholecystitis with typical pain, no ascites, and normal vascular flow. Platelets declined transfusion dependency but not refractoriness. Based on these results, sepsis was diagnosed. Antibiotic treatment was escalated to meropenem/vancomycin, and bacterial, fungal, and viral infection screenings were performed without positive results.

The patient refused a liver biopsy. Without improvement on anti-infective therapy, bilirubin rose to 17.8 mg/dl, and moderate ascites appeared on day 38. A day later, the patient deteriorated rapidly with respiratory failure, renal failure, new pneumonia, and bilirubin 31 mg/dl. Despite intensive care treatment, she died on day 41 with apparent MOF. No other signs of GvHD (skin/gut) appeared during the treatment.

Pathological examination of the liver showed central veins with constriction to complete obliteration, typical of VOD/SOS, without signs of GvHD or infection (Fig. 1).

**Fig. 1:**
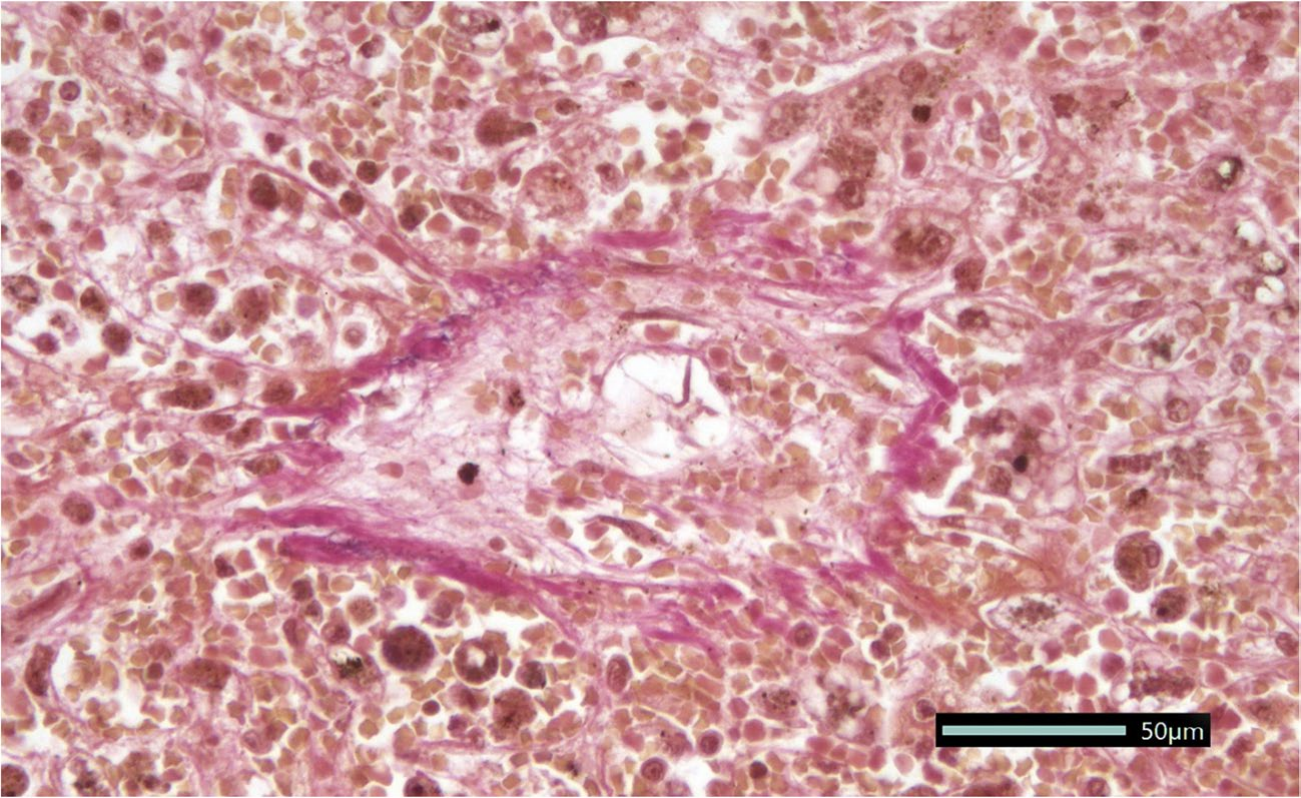
Postmortem light microscopy of the central vein of the liver with substantial sinusoidal obliteration (VOD/SOS) and accumulation of red blood cells (EvG trichrome stain)

This patient showed signs of infection and sepsis, with features of severe VOD/SOS per EBMT criteria on day 33. Rapid diagnosis and management of VOD/SOS are often delayed by missing specific clinical signs, unfulfilled diagnostic criteria, competing/overlapping diagnoses like GvHD or sepsis or critically ill patients and fear for treatment-related side effects like bleeding. If bleeding is a side effect of treatment or caused by the hepatic failure itself remains elusive in the lack of randomized trials.

Since VOD diagnosis is often missed [3] or misdiagnosed [4] like in our case, we recommend preemptive treatment of any suspected case, guided by EBMT criteria, until symptoms resolve or there is proof of other causes and early biopsy (transcutaneous or transjugular), for histological diagnosis. Additional diagnostic criteria, such as imaging with CT/MRT or ultrasound/elastography [5] or, to a less extend, biomarkers show high sensitivity without proof of specificity so far. Prospective evaluation of different diagnostic and grading criteria [6] with higher specificity, allowing an earlier diagnosis, are needed.

## References

[1] Richardson PG, Smith AR, Kernan NA, Lehmann L, Soiffer RJ, Ryan RJ, Tappe W, Grupp S (2020). Pooled analysis of Day 100 survival for defibrotide-treated patients with hepatic veno-occlusive disease/sinusoidal obstruction syndrome and ventilator or dialysis dependence following haematopoietic cell transplantation. Br J Haematol.

[2] Mohty M, Malard F, Abecassis M, Aerts E, Alaskar AS, Aljurf M, Arat M, Bader P, Baron F, Bazarbachi A, Blaise D, Ciceri F, Corbacioglu S, Dalle JH, Dignan F, Fukuda T, Huynh A, Masszi T, Michallet M, Nagler A, NiChonghaile M, Okamoto S, Pagliuca A, Peters C, Petersen FB, Richardson PG, Ruutu T, Savani BN, Wallhult E, Yakoub-Agha I, Duarte RF, Carreras E (2016). Revised diagnosis and severity criteria for sinusoidal obstruction syndrome/veno-occlusive disease in adult patients: a new classification from the European Society for Blood and Marrow Transplantation. Bone Marrow Transplant.

[3] Ma C, Brunt EM (2016). Terminal hepatic venule injury in liver biopsies of allogeneic haematopoietic stem cell recipients—a study of 63 cases. Histopathology.

[4] Ruggiu M, Bedossa P, Rautou PE, Bertheau P, Plessier A, Peffault de Latour R, Robin M, Sicre de Fontbrune F, Pagliuca S, Villate A, Xhaard A, Socié G, Michonneau D (2018). Utility and safety of liver biopsy in patients with undetermined liver blood test anomalies after allogeneic hematopoietic stem cell transplantation: a monocentric retrospective cohort study. Biol Blood Marrow Transplant.

[5] Chan SS, Colecchia A, Duarte RF, Bonifazi F, Ravaioli F, Bourhis JH (2020). Imaging in hepatic veno-occlusive disease/sinusoidal obstruction syndrome. Biol Blood Marrow Transplant.

[6] Cairo MS, Cooke KR, Lazarus HM, Chao N (2020). Modified diagnostic criteria, grading classification and newly elucidated pathophysiology of hepatic SOS/VOD after haematopoietic cell transplantation. Br J Haematol.

